# Molecular pathological classification of colorectal cancer—an update

**DOI:** 10.1007/s00428-024-03746-3

**Published:** 2024-02-06

**Authors:** Philip D. Dunne, Mark J. Arends

**Affiliations:** 1 Patrick G. Johnston Centre for Cancer Research, Queens University Belfast, Belfast, Northern Ireland BT8 7AE UK; 2 Cancer Research UK Scotland Institute, Garscube Estate, Glasgow, G61 1QH UK; 3 Edinburgh Pathology & Cancer Research UK Scotland Centre, Institute of Genetics & Cancer, University of Edinburgh, Crewe Road, Edinburgh, EH4 2XR UK

**Keywords:** Colorectal cancer, Molecular pathological classification, Hereditary

## Abstract

Colorectal cancer (CRC) has a broad range of molecular alterations with two major mechanisms of genomic instability (chromosomal instability and microsatellite instability) and has been subclassified into 4 consensus molecular subtypes (CMS) based on bulk RNA sequence data. Here, we update the molecular pathological classification of CRC with an overview of more recent bulk and single-cell RNA data analysis for development of transcriptional classifiers and risk stratification methods, taking into account the marked inter-tumoural and intra-tumoural heterogeneity of CRC. The importance of the stromal and immune components or tumour microenvironment (TME) to prognosis has emerged from these analyses. Attempts to remove the contribution of the tumour microenvironment and reveal neoplastic-specific transcriptional traits involved identification of the CRC intrinsic subtypes (CRIS). The use of immunohistochemistry and digital pathology to implement classification systems are evolving fields. Conventional adenoma versus serrated polyp pathway transcriptomic analysis and characterisation of canonical LGR5+ crypt base columnar stem cell versus ANXA1+ regenerative stem cell phenotypes emerged as key properties for improved understanding of transcriptional signals involved in molecular subclassification of colorectal cancers. Recently, classification by three pathway-derived subtypes (PDS1-3) has been developed, revealing a continuum of intrinsic biology associated with biological, stem cell, histopathological, and clinical attributes.

## Introduction

Colorectal cancer (CRC) is the fourth most common cancer in men and women [1]. Most CRC, around 70–80%, are sporadic, while around 20–30% of CRC have a hereditary component, due to either uncommon or rare, high-risk, genetic tumour syndromes, such as Lynch Syndrome (LS) (3–4%) and familial adenomatous polyposis (FAP) (∼ 1%) amongst others [2, 3], or more common but low-risk alleles identified by genome-wide association studies (GWAS) [4, 5]. Only 1–2% of CRC cases arise from inflammatory bowel diseases [6].

## Molecular pathways and classification

**Fig. 1:**
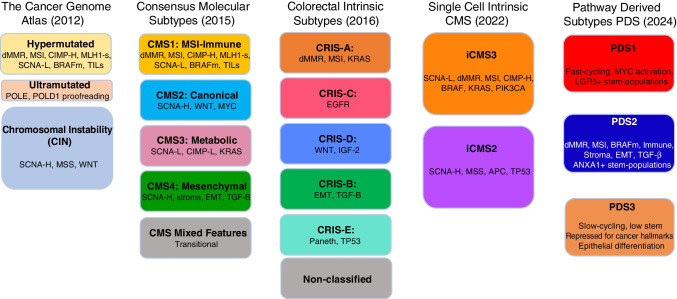
Diagrammatic summary of colorectal cancer molecular pathology classification systems. The Cancer Genome Atlas (TCGA), published in 2012, used a predominantly DNA-based classification, splitting colorectal cancers (CRC) into a large group (84%) with chromosomal instability (CIN), 13% hypermutated CRC due to deficient mismatch repair (dMMR) that causes microsatellite instability (MSI), and 3% ultramutated CRC due to proofreading exonuclease domain mutations in the two polymerases POLE and POLD1. The consensus molecular subtype (CMS) classification, published in 2015, used bulk RNA sequences to classify CRC into 4 major groups, CMS1–CMS4, with a residual ‘Mixed Features’ or transitional group. CMS1 correlated strongly with the TCGA hypermutated group. The large TCGA CIN group splits into 3 CMS groups—CMS2: canonical, CMS3: metabolic, and CMS4: mesenchymal with the features shown. The Colorectal Intrinsic Subtypes (CRIS) classification, published in 2016, separated CRC epithelial neoplastic cells (without stromal and immune components) into 5 subtypes, CRIS-A to CRIS-E. Most CMS1 and CMS3 cancers fell into CRIS-A (some CMS1 were in CRIS-B); CMS2 cancers were found within CRIS-C, CRIS-D, and CRIS-E; CMS4 cancers split into CRIS-B, CRIS-C, CRIS-D, and CRIS-E. The single-cell intrinsic consensus molecular subtype (iCMS) classification of CRC, published in 2022, based on single-cell transcriptomes identified a transcriptomic dichotomy of malignant cells, resulting in two intrinsic subtypes, iCMS2 and iCMS3, that refined the earlier CMS classification. Most CMS2 and CMS3 CRCs have iCMS2 and iCMS3 epithelium, respectively, whereas iCMS3 contains the dMMR/MSI cancers and one-third of microsatellite-stable (MSS) tumours. The Pathway-Derived Subtypes (PDS) classification of CRC, published in 2023, puts colorectal cancers into 3 subtypes, PDS1–PDS3, with a small residual PDS Mixed group, based on Gene Ontology inferred pathway activation patterns, revealing a continuum of features associated with biological pathways, stem cell populations, morphological/histopathological characteristics, and clinical attributes. Abbreviations: dMMR, deficient mismatch repair; MSI, microsatellite instability; MSS, microsatellite stability; CIMP, CpG island methylator phenotype (-H, high or -L, low); SCNA, somatic copy number alteration (-H, high or -L, low); MLH1-s, silencing of MLH1 protein expression by promoter hypermethylation; *BRAF*m, *BRAF* mutation; TILs, tumour infiltrating lymphocytes; EMT, epithelial-mesenchymal transition; CBC, LGR5+ crypt base columnar stem cell; RSC, ANXA1+ regenerative stem cell; SMI, Stem Maturation Index

In 2016, we provided an overview and integration of the molecular classification of CRC [7], emphasising that it is not a homogenous disease, but can be classified into different subtypes, characterised by specific molecular features discovered over the preceding three decades. At the genomic level, despite a very wide range of individual gene alterations, CRC shows two major mechanisms of genomic instability: chromosomal instability (CIN) and microsatellite instability (MSI). Those CRC with chromosomal instability are most common (around ∼ 84% of sporadic CRC) and are characterised by gross changes in chromosome number and structure including deletions, gains, amplifications, translocations, and other often complex chromosomal rearrangements. These are often detectable as a high frequency of DNA somatic copy number alterations (SCNA), which are a hallmark of most tumours that arise by the adenoma-carcinoma sequence [8]. Other studies have associated CIN with inactivating mutations or losses in the adenomatous polyposis coli (*APC*) tumour suppressor gene, which occur as an early event in the development of bowel neoplasia in this sequence, and/or inactivation of *TP53*, the guardian of the genome, or other pathway members [2, 9, 10]. The second group (around ∼ 13–16% of sporadic CRC) is hypermutated and shows MSI due to defective DNA mismatch repair (MMR), often associated with wild-type *TP53* and a near-diploid pattern of chromosomal stability [11–13]. Furthermore, MSI CRC often shows CpG island methylation phenotype (CIMP), which is a feature that induces epigenetic instability by promotor hypermethylation and silencing of a range of tumour suppressor genes, including *MLH1*, one of the MMR genes [14]. The integrated molecular analysis by The Cancer Genome Atlas project in 2012 [15] confirmed this largely DNA-based classification of CRC into two major groups of MSI CRC (∼ 13–16%) and (2) CIN CRC (∼ 84%) (Fig. 1). Our previous review [7] also briefly covered CRC classification at the transcriptomic level by the Consensus Molecular Subtypes (CMS) Consortium (2015) [16], which analysed CRC bulk RNA expression profiling data from multiple studies to describe four major CMS groups, with a residual mixed group (Fig. 1).

While the two-class DNA-based model (CIN and MSI) identified by genomic instability identifies tumour groups that are clearly distinct from each other in terms of their mutational and copy number alterations, there is an increasing recognition that there remains significant heterogeneity within tumours that are robustly classified as CIN or MSI. The presence and extent of this inter-tumour heterogeneity has been the focus of numerous studies since our previous review, each of which has provided new classification models that aim to capture transcriptional signalling and clinical phenotypes of interest. Furthermore, there have been a number of studies that aim to identify and characterise the factors underpinning these variations, using modern methodologies that have enabled further insight into the molecular and histological features that underpin the intra-tumoural heterogeneity that exists within individual tumours.

## History of transcriptional classifications

The clinical value of using transcriptional data as the basis for molecular subtyping in cancer was demonstrated more than two decades ago, by a series of seminal studies in breast cancer [17, 18]. Breast cancers were aggregated into biologically similar subtypes that aligned with prognosis and some previously defined clinical attributes. This pioneering work led to the development of tools like MammaPrint, PAM50, and OncotypeDx to provide information of a patient’s risk of relapse and potential response to chemotherapy.

In the years that followed, the use of transcriptional subtyping in colorectal cancer was largely confined to the development of risk stratifiers that could be used to identify patients with highest risk of disease relapse following surgery in stage II CRC, culminating in the development of a number of FDA-approved diagnostic tests and prognostic assays, such as ColDx and OncotypeDx [19, 20]. However, more recent studies have shown that the biological traits associated with relapse in one subtype can be quite different in another, a point that weakens the value of these general risk stratification tools and supports molecular stratification as a primary assessment of the correlations between transcriptional data and clinical outcomes [21].

## Molecular subtyping of CRC 2010–2016

One of the primary goals of molecular subtyping is the generation of molecular biomarkers in cancer that can be used to stratify tumours according to clinical risk groups or biological subtypes, which in turn provide improved understanding of signalling cascades that underpin tumour development and treatment response. As described in our previous review [7], the first landmark classification model in CRC that used multi-omic molecular information was published in 2012 as part of The Cancer Genome Atlas (TCGA) network project [15]. This study utilised mutational, epigenetic, mRNA, and miRNA information to identify molecular subtypes that strongly aligned with the CIN and MSI dogma, with additional substratification of the MSI group based on the level of mutational burden. Highly mutated CRC (∼ 16%) was split into two major groups: (1) hypermutated cancers (∼ 13%) with microsatellite instability (MSI) due to defective mismatch repair (dMMR) or (2) ultramutated cancers (∼ 3%) with DNA polymerase epsilon (POLE) exonuclease domain mutations that inactivate the proofreading function. In contrast, CRC with lower mutation rates (∼ 84%) that were non-hypermutated, microsatellite stable (MSS) cancers, with a high frequency of DNA somatic copy number alterations due to CIN, commonly showed mutations or deletions in *APC*, *TP53*, *KRAS*, *PIK3CA*, and *SMAD4* (Fig. 1) [8, 15, 22].

In the same time period as the TCGA study was published, numerous other studies proposed molecular subtypes of CRC, primarily defined using transcriptional information (at least 5 of them). Remarkably, despite each of them using similar and, in some cases, the same data, there appeared to be little agreement between the genes and biomarkers that underpinned each approach. The reasons for this may be attributed to both technical variations that can undermine cross-platform comparisons of individual gene-level biomarkers and also the different bioinformatic approaches employed by each study for identification, characterisation, and final classifier deployment in these datasets.

To provide clarity to the field and to enable the development of a unified approach to molecular subtyping, an international consortium was assembled that included many of the groups that were behind the development of these individual approaches. This consortium, spanning more than 15 institutes and utilising data from more than 5000 tumour samples, sets out to identify concordance across the previously reported subtypes, leading to the establishment of a new paradigm in the field, the consensus molecular subtypes (CMS), comprising four major groups CMS1-4 (described briefly in our previous review) [16] (Fig. 1). The emergence of this consensus approach in CRC classification enabled the field to have a stable reference point across pre-clinical and clinical research. Numerous studies quickly deployed the CMS approach on clinical samples, to enable retrospective alignment with outcome and response to treatment modalities and to pre-clinical models in the hope of developing new understanding and potential therapeutics for these newly defined transcriptional subtypes.

## Role and contributions of the stroma to transcriptome and prognosis

Previously, De Sousa E Melo et al. [23] demonstrated that although different prognostic signatures generally consist of non-overlapping sets of genes, they almost always identify the same group of poor-prognostic cases, suggesting that while individual genes are redundant, it is the most distinctive overall biological features that are associated with prognosis. In line with this data, multiple molecular subtyping studies in CRC have identified levels of fibroblast infiltration and genes specifically originating from cancer-associated fibroblasts (CAFs) as key factors in disease relapse [24–26]. The influence of the tumour microenvironment (TME) on the CMS classification and wider transcriptional signalling system was identified by numerous studies [24, 25], and the potential implication for intratumoural heterogeneity in diagnostic samples due to multi-regional sampling was demonstrated [27].

In an attempt to remove the contribution of the tumour microenvironment and reveal neoplastic-specific transcriptional traits, the CRC intrinsic subtypes (CRIS) were developed using profiling of tumour xenografts that filtered out signals from stromal and immune components [28], which in turn resulted in increased stability of classification. The CRIS approach identified five new subtypes, CRIS-A to CRIS-E (Fig. 1). CRIS-A was associated with MSI or *KRAS* mutations and mucinous features. CRIS-B tumours showed TGF-B pathway activity with EMT and a poor prognosis. CRIS-C cancers had elevated EGFR signalling with sensitivity to EGFR inhibitors. CRIS-D tumours showed WNT pathway activation with *IGF-2* gene overexpression or amplification. CRIS-E had a Paneth cell-like phenotype with *TP53* mutations. This CRIS subtyping successfully categorised independent primary and metastatic CRC datasets.

However, while these molecular studies identified the transcriptional consequences of these variations, the fundamental role played by the stromal and immune components in CRC when classifying tumours into clinically valuable categories was clearly defined by Jass et al. in the late 1980s [29], who presented a histological system that outperformed Dukes’ staging for predicting clinical outcomes of rectal cancers. This system utilised information equivalent to T and N status, alongside information about the presence and extent of lymphocytic infiltration and epithelial infiltration at the tumour margins, features reminiscent of the traits that are most prominent in CMS1 and CMS4 tumours.

While the promise of the precision medicine era in CRC was heralded by the development of CMS and other tools, the absence of a large clinical impact may be seen as a failure over the last decade. There are numerous underlying reasons for this; however, in the next sections, we focus on the incompatible nature of molecular stratification within the turnaround times required for diagnostic decision-making.

## Rapid turn-around CMS classification and emergence of morphology, immunohistochemistry (IHC), and image-based surrogates

Currently, molecular analysis of CRCs for a timely pathology report often involves determination of proficient or deficient mismatch repair status, mostly by MMR immunohistochemistry, using either the 2-antibody [30] or conventional 4-antibody approach. Some laboratories perform MSI testing on tumour DNA, as an alternative to MMR immunohistochemistry, or in combination with it to resolve staining discrepancies [3]. For metastatic CRC, mutational analysis of all *RAS* genes may be performed when oncologists are considering anti-epidermal growth factor receptor (EGFR) therapy. CRCs with the *BRAF* V600E substitution may show aggressive behaviour and could be treated with combined BRAF and EGFR inhibition. Some CRCs have overexpression of the *HER2* oncogene that may be analysed by immunohistochemistry and/or in situ hybridisation as they may respond to appropriate targeted therapy [22, 31].

**Fig. 2:**
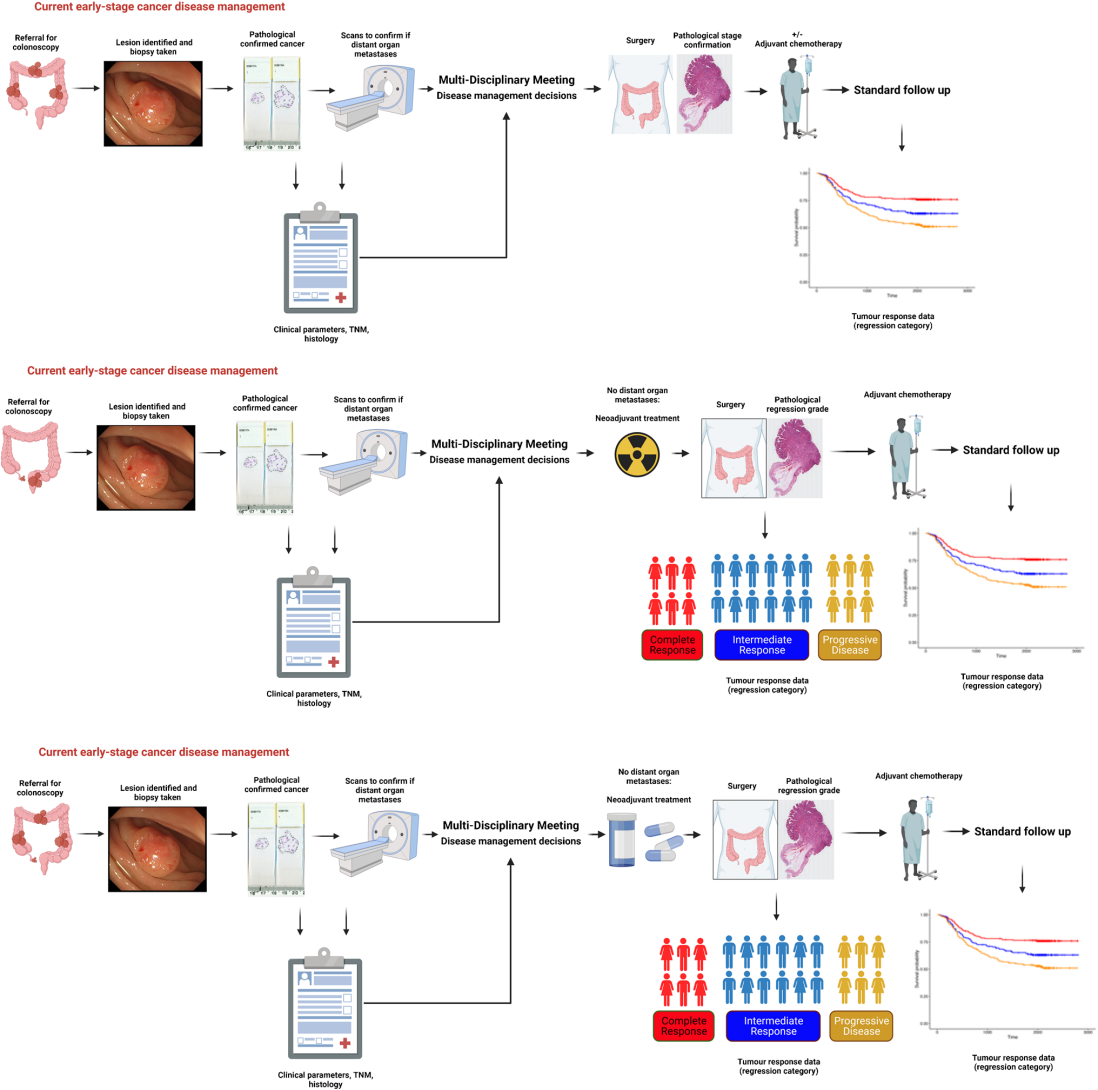
Typical standard-of-care pathway for early stage (stage I–III) localised colonic or rectal cancers. Following the patient coming to the pathway due to bowel symptoms, screening, or referral, endoscopic biopsies are taken for histopathological diagnosis (also available for some biomarker testing, such as for *KRAS* mutation analysis in certain circumstances) and combined with radiological scans to determine likely cancer stage, with the key cancer and patient features being discussed at a multi-disciplinary team (MDT) meeting for making appropriate management decisions, including treatment of the patient, which may involve resection and/or adjuvant chemotherapy (upper panel) and/or neoadjuvant radiotherapy (middle panel) and/or neoadjuvant chemotherapy (lower panel), with tumour response/regression analysis made by histopathological assessment of resection specimens

Stratification of cancer patients into specific treatment groups, based on the molecular pathological changes of their tumours, has the potential to improve patient outcomes by delivering the right drug to the right patient. Development of predictive biomarkers for clinical use has relied largely on evaluation using low-throughput methods on single-gene status, for example, with *KRAS* mutational status in CRC as a predictive marker of resistance to EGFR inhibition. The implementation of prospective molecular stratification in randomised controlled trials (RCTs) such as FOxTROT [32] has demonstrated the feasibility of a rapid turnaround (within a week was the aim) for DNA extraction and *RAS* mutation analysis by pyrosequencing. In line with this requirement, there have been highly accurate CE-marked in vitro diagnostic device tools developed to deliver rapid-turnaround and easy to interpret results [33–35]. However, in contrast to single-gene biomarkers, more complex multi-gene and multi-omics classifiers can lead to a significant time lag between tissue processing, molecular profiling, data analysis, and result availability to the clinician. The ‘standard-of-care’ pathway for early stage (stage I–III) localised colonic or rectal cancer is shown in Fig. 2, in which radiological scans and tissue samples taken during endoscopy can be assessed histopathologically to provide the diagnosis and staging of the cancer within the clinically acceptable timeline. As neo-adjuvant response rates improve, the tissue obtained at the initial diagnosis is, in some cases, the only pathological material retrieved from the patient and hence the only material on which to carry out molecular profiling. With advances in molecular profiling technologies, the ability to successfully extract meaningful molecular information from even small, degraded samples increase; however, the time lag for feeding this information back to clinicians for discussion at a multidisciplinary team (MDT) meeting remains a critical issue. Therefore, if the potential patient benefit of this molecular stratification is ever to be realised, this process needs to be moved into rapid-turnaround prospective stratification to fit with the clinical timeline. Many have attempted to link CRC morphological patterns with molecular features with varying degrees of success. However, Budinska et al. (2023) have suggested that the main molecular signals align with characteristic morphological patterns seen in CRC and they examined the extent to which morphotype heterogeneity impedes prognostic and predictive expression-based classifiers [36].

## IHC approach to CMS classification

To circumvent the need for molecular profiling and to construct a CMS classification approach that can be deployed in current diagnostic pathology laboratories, Trinh et al. [37] developed a five-marker IHC panel (FRMD6, ZEB1, HTR2B, CDX2, and cytokeratin) that works alongside standard MSI/dMMR testing to deliver a practical classification tool with 87% concordance with the ‘gold-standard’ transcriptomic CMS classification. This system utilised dMMR/MSI status to define CMS1, with remaining MSS cancers being classified into two classes using four of the IHC markers, either an epithelial (CMS2/3 combined) or mesenchymal (CMS4) subtype, with epithelial content being normalised using pan-cytokeratin IHC. The authors acknowledged the lack of separation between the epithelial classes, CMS2 and CMS3, as a limitation of this initial approach, which is driven by distinct biological signalling in the original CMS study. However, this IHC approach to CMS classification has not achieved widespread usage. In addition, as noted in other transcriptomics-based studies, the presence and extent of intratumoural heterogeneity and lack of standard biopsy sampling protocols can potentially undermine classification.

## Digital pathology and image-based H&E approaches

The emergence and recent rapid acceleration of the field of digital pathology have been facilitated by the ongoing development of tools like QuPath and Halo [38] that support the generation of methodologies reliant on deep learning and AI, so too will opportunities to rapidly classify diagnostic samples in parallel with pathologist assessment. Development of digital pathology tools has enabled histology-based classification systems to be developed and applied to routine diagnostic H&E samples. Given the strong influence that the tumour microenvironment plays in CMS classification, the emergence of robust image-based classification tools represents a rapid and cost-effective way for upfront decision-making in clinical trials. Using ‘ground-truth’ transcriptional CMS calls from > 1200 tumours with sample-matched H&Es and transcriptional data from both tumour resections and pre-treatment biopsies assembled within the S:CORT consortium, Sirinukunwattana et al. [39] developed an image-based CMS (imCMS) deep learning classifier that could accurately call the four CMS classes when deployed on independent samples (AUC = 0.84 in TCGA samples, and AUC = 0.85 in rectal biopsies). While the headline figures for concordance with transcriptional CMS calls appear similar to the IHC approach, the value of the imCMS method was that it could call each of the four discrete CMS classes, as opposed to combining CMS2 and CMS3 and segregating these from CMS4.

In addition, this imCMS approach did not require parallel MSI/dMMR testing and IHC staining, as it was designed to be performed on diagnostic H&E images. More importantly, alongside the overall sample-level classification, the image-based approach provides an insight into tile-level classification that make up this overall call, enabling a more accurate spatial assessment of the presence and extent of intratumoural heterogeneity in individual samples, an issue that had previously been reported through multi-regional transcriptional assessments.

## Single-cell intrinsic CMS (iCMS) classifier

As single-cell sequencing has become more routine in tumour profiling studies, the emergence of molecular classification from these data types has the potential to add more granularity to those using bulk tumour data. By using data derived from ~ 50,000 epithelial cells, Joanito et al. [40] developed the single-cell intrinsic CMS (iCMS) classification model, which identified two epithelial classes with distinct gene expression, transcriptional factor activity, and genomic profiles. In the single-cell data, the authors reported that the iCMS2 class was associated with SCNA/copy number variation (CNV) across many chromosomal regions, whereas iCMS3 displayed limited uniformity in CNVs (Fig. 1). In contrast, almost all MSI tumours were classified as iCMS3, and given this association, these tumours were also associated with mutational burden, CIMP, right-sidedness, and mucinous tumours. The authors demonstrated that this new two-class iCMS system could be combined with the bulk CMS four-class approach, to separate CMS4 tumours into new prognostic groups according to iCMS2 (better outcome) or iCMS3 (worse outcome). Remarkably, although the new iCMS system was based on intrinsic epithelial traits, when applied to bulk tumour data, the iCMS classifier was unable to find any distinct underlying biology within the epithelial-rich CMS2 (that accounts for ~ 40% of CRCs) and CMS3 subtypes, which were almost exclusively assigned to iCMS2 and iCMS3, respectively. CMS1 tumours were almost exclusively assigned as iCMS3. Overall, iCMS3 tumours were more likely to be associated with *BRAF*, *KRAS*, and *PIK3CA* mutations, whereas iCMS2 tumours were associated with mutations in *APC* and *TP53*. The authors propose a final model, termed the intrinsic-MSI-fibrosis (IMF) system, as the most informative as it considers the iCMS classification, microsatellite instability status, and levels of CAF-related fibrosis.

## Single-cell polyp progression

**Fig. 3:**
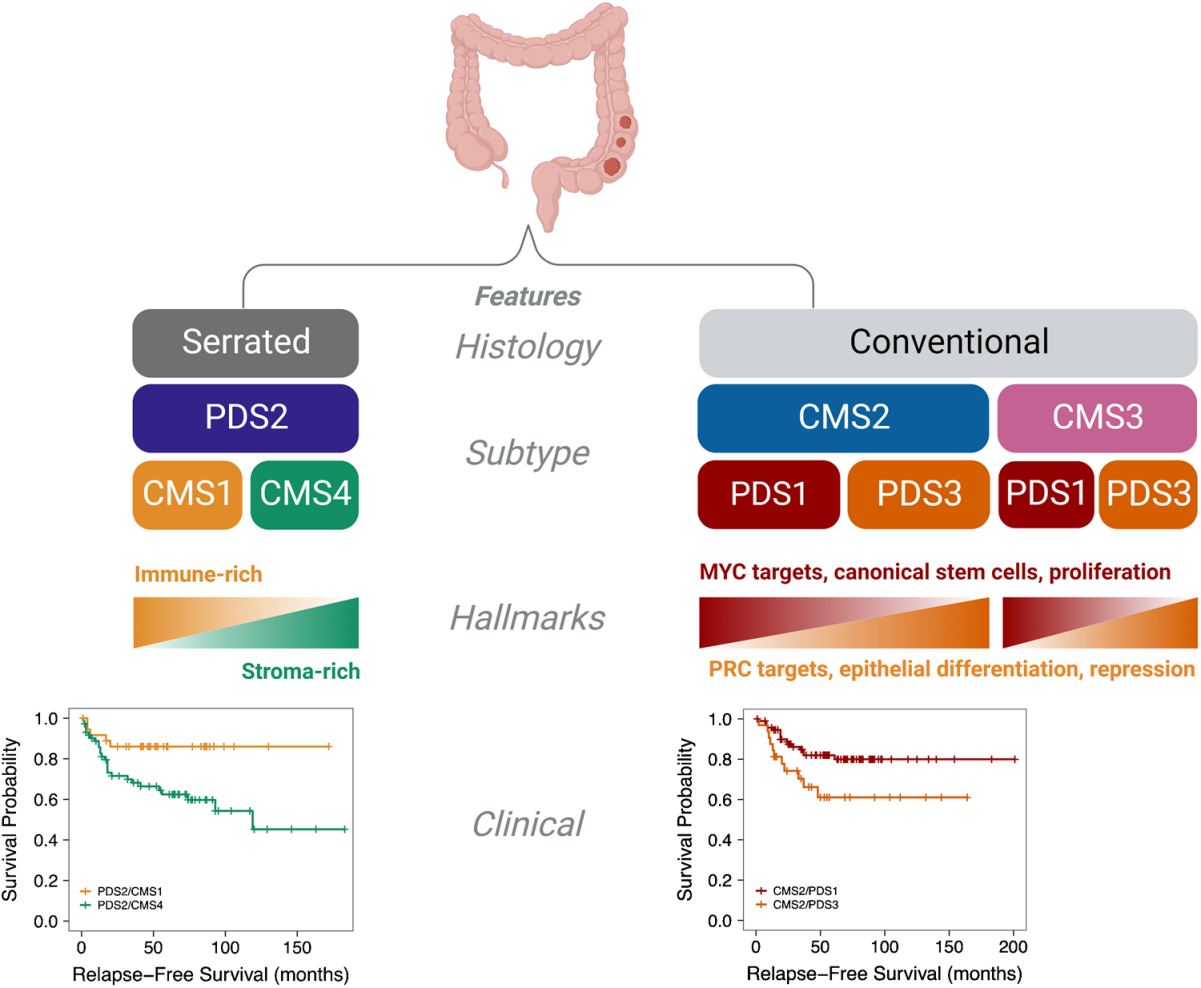
Comparison of colorectal serrated polyp (‘Serrated’) versus conventional adenoma (‘Conventional’) pathway progression to cancers in terms of cancer histology, cancer subtype (CMS1/4 and PDS2 versus CMS2/3 and PDS1/3), selected cancer hallmarks (immune-rich/stroma-rich versus MYC targets, canonical stem cells, proliferation, PRC targets, epithelial differentiation, and repression), and clinical relapse-free survival differences

**Fig. 4:**
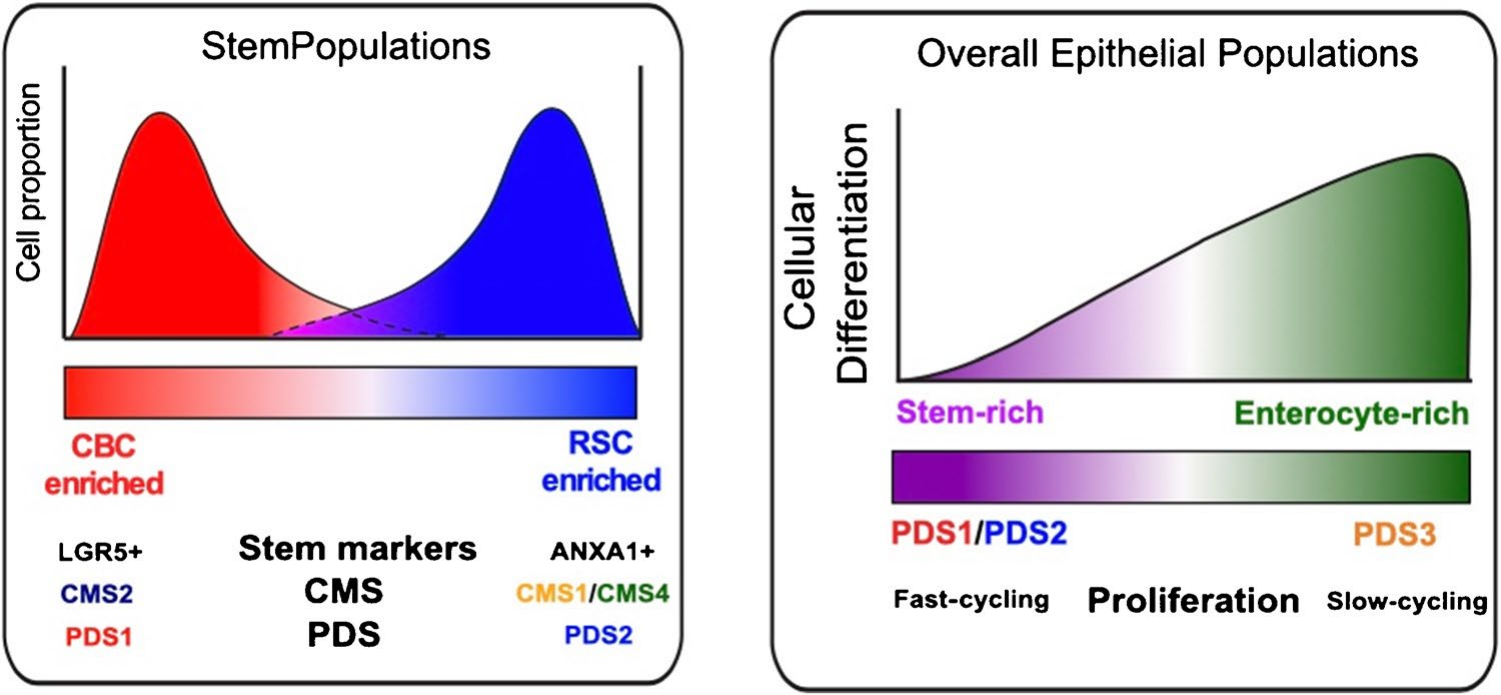
Transcriptomic analysis of stem cell populations in CRC showed variable populations of both LGR5+ /ANXA1− crypt base columnar (CBC) stem cells and LGR5-/ANXA1+ regenerative stem cells (RSC), reflecting stem cell plasticity that can respond adaptively to acute selective pressures, and this admixture can be assessed using a transcriptional molecular tool to assess the Intestinal Stem Cell Index (ISC), with CMS2 and PDS1 groups enriched for LGR5+ CBC stem cells, whereas CMS1/CMS4 and PDS2 groups are enriched for ANXA1+ RSC. Combining PDS and stem cell analyses showed that both LGR5+ CBC and ANXA1+ RSC were fast-cycling and abundant in stem-rich PDS1 and PDS2, whereas the PDS3 group was slow-cycling and stem-poor, containing more enterocytic differentiated cells as shown by the Stem Maturation Index (SMI)

While the iCMS proposed a set of epithelial classes that are evident in cancer, a number of recent studies have used similar single-cell technologies from 62 patients, across discovery and validation cohorts within the COLON MAP study, to provide more insight into the cell states within conventional adenomas and serrated polyps, alongside the cancers that arise from these developmental pathways [41]. This work confirmed many of the previously defined molecular associations associated with these classes of precancers, including associations of *APC* abnormalities with the conventional adenoma pathway and *BRAF* alterations with serrated polyps. This work also confirmed the elevation of LGR5+ cell populations (described later in more detail) in conventional adenomas compared with normal; however, in serrated polyp lesions, no such elevation was noted, with the authors proposing a process associated with metaplasia and loss of expression of the homeobox transcriptional regulator CDX2. The authors proposed that these changes are driven, in part, as a wound-healing response with a regenerative stem-like phenotype (Fig. 3, 4).

A key finding in this study was that in serrated lesions, the presence of cytotoxic cells (CD8+ T cells, NKs, and gdT cells), alongside the activation of an antigen processing and presentation gene signature, was significantly elevated compared with conventional adenomas, similar traits that were elevated in MSI tumours compared to MSS. Importantly, however, these traits were all observed prior to the onset of increased mutational burden (as a result of dMMR/MSI), providing evidence of triggers that drive activation of adaptive immunity in precancerous lesions that appear to be independent of dMMR-driven hypermutation. The authors utilised a set of *BRAF*-driven (*Lrig1 CreERT2*/+ ; *Braf* LSL-V600E/+) and *KRAS*-driven (*Lrig1 CreERT2*/+ ; *Kras* LSL-G12D/+) mouse models of serrated lesions to identify that it is the non-stem differentiated epithelial lineages that give rise to the immune activated environment.

Becker et al. [42] recently defined a continuum of biological signalling, using single-cell RNA sequencing and chromatin profiling, that aligned with the changes in cellular states during normal-precancer-cancer progression in CRC using a cohort of 48 polyps, 27 normal tissues, and 6 cancers collected from 15 patients. Importantly, these cases were disproportionately derived from patients with familial adenomatous polyposis (FAP), with 8 FAP and 7 non-FAP patients. The authors identified a clear elevation in the proportions of components of a cancer-associated TME during normal to cancer progression, namely, increased Tregs, exhausted T cells, pre-CAFs, and mature CAFs.

These TME changes track in parallel with an increase in stem-like epithelial cells from normal to precancer; however, this significant trend did not follow through in the cancer samples, which split evenly into groups with either extremely high stem-like signalling or a group with stem-like epithelial cells equivalent to unaffected colonic tissue. This latter group was suggestive of an alternative progression pathway from precancer to cancer in this subset of cases; however, as indicated above, this may primarily apply to FAP-related cases.

## Stem cell classifications and plasticity

Given that epithelial cells are continuously lost due to apoptosis and shedding, the existence of a colonic stem cell population that gives rise to, and replenishes, all epithelial cells lining the intestinal mucosa has been long established. While these stem cells were thought to be located towards the base of the crypts of Lieberkühn in the small intestine, the subsequent discovery and characterisation of colonic crypt base columnar cells (CBCs), and the rapidly proliferating self-renewal properties they displayed, provided a critical explanation for the source and maintenance of many of the key phenotypes observed in colorectal cancer. As studies on CBCs increased, the identification of key selective biomarkers, like LGR5+ [43] and the ability of these stem cells to serve as the ‘cell-of-origin’ for tumourigenesis following inactivation of *APC* [44], further reinforced this hypothesis.

Although LGR5-positivity provides a marker of CBC stem cells, there have been numerous reports of how LGR5-negative cells can also give rise to neoplastic lesions, particularly within inflammatory, regenerative, or desmoplastic stromal environments [45, 46]. The association between an LGR5-negative cell-of-origin and stromal/inflammatory lesions aligns well with the findings from the single-cell polyp study mentioned earlier [41] that described the dominance of a differentiated regenerative-like metaplasia stem population in serrated polyps. In parallel, recent studies have highlighted that these non-canonical LGR5-negative stem populations may also be the drivers of CRC dissemination, tumour budding, and relapse [47], and while they may account for a small population in primary tumours, they are strongly enriched in metastatic lesions [48].

LGR5 negativity has been associated with inflamed tumours, and elegant recent work has demonstrated how stem cells and, indeed lesions overall, can shift between these LGR5-positive CBC and LGR5-negative ANXA1+ regenerative stem cell (RSC) states, defined as plasticity, as they adapt and respond to changes in microenvironmental conditions (Fig. 4) [46]. At the same time, using heterotypic organoid co-culture models, another recent study revealed that the steps involved in the regulation of this stem cell plasticity can be attributed to both cell-intrinsic and microenvironmental signalling [49], focusing on colonic stem cells (CSC), their regenerative populations described as revival colonic stem cells (revCSC), alongside identification of a distinct set of hyperproliferative colonic stem cells (proCSC).

These studies, using both bulk and single-cell technologies, have revealed the presence and extent of heterogeneity in stem cell populations within CRC, providing a more detailed assessment of the dynamics and consequences of the inter- and intra-cellular signalling networks that are ongoing within the heterogeneous milieu of lineages within a tumour mass. Importantly, however, these studies have used different terminology to describe each of the possibly overlapping stem populations, meaning that there remains a need for detailed assessment of each of these biomarkers in an agreed way in order to produce more consistent nomenclature.

## Limitations of gene-based classifiers versus pathway-based classifiers

The molecular subtyping approaches described thus far, using both bulk and single-cell data, rely on methodologies defined by the early breast cancer subtyping work of the late 1990s and early 2000s, where individual gene-level expression data from microarray or RNAseq served as the template for aggregating tumours into similar subgroups. In contrast, it is well understood that pathway-level data, where genes are arranged into experimentally validated pathway signatures to represent important biological signalling pathways, provides a quick and reproducible way to test associations between groups of samples according to a broad range of molecular mechanisms and phenotypes. Given the biological value that pathway-level data provides, almost all molecular subtyping studies may use collections of such Gene Ontology signatures, like the Molecular Signature Database (MSigDB), to identify significant associations between these pathways and their identified subtypes [50]. Significantly elevated signalling can then be used as the hallmark features in each subtype compared to the others; as exemplified by cell cycle activation and signalling in both WNT and MYC targets in CMS2, metabolic signalling pathways in CMS3, and TGF-β activation and EMT signalling in CMS4 [16]. Based on these successes, gene-level discovery followed by pathway-level characterisation represents a more widely applicable approach.

Given that the end goal of many subtyping development studies is an eventual alignment and characterisation with important biological phenotypes in each subtype, we proposed a new method that changes the sequence of this stepwise approach, with the aim of providing a closer link with molecular mechanisms and clinical phenotypes. This pathway-level approach should replace the initial gene-level clustering by directly using these Gene Ontology and biological pathway signatures as the basis for grouping samples.

## Pathway-derived subtypes

The first step in this alternative class discovery approach was to convert all our existing gene-level data cohorts into pathway-level scores prior to subtype discovery, across ~ 2000 signatures associated with biological processes contained within these databases to generate a matrix of 640,000 + combinations of biological phenotypes. When clustering is performed, using the methods in the same way as other gene-level studies, three pathway-derived subtypes (PDS) in CRC were identified, where PDS1 (26%), PDS2 (31%), PDS3 (30%), and a smaller more heterogeneous residual ‘mixed’ group that accounted for ~ 13% of tumours across the CRC cohorts (Fig. 1) [51].

Comparing the PDS and CMS classifications of the same data revealed granularity within the largest tumour subtype defined as epithelial-rich with uniform signalling attributes in the original CMS study, CMS2 group, which was now split almost equally into two highly distinct transcriptional subtypes, PDS1 and PDS3. At the same time, as identifying granularity in the epithelial-rich subtypes, the PDS approach found that the inflammatory/stromal CMS1/CMS4 subtypes were combined within a single subtype, PDS2 (Fig. 4) [51].

Remarkably, despite the clearly distinct transcriptional landscapes observed according to PDS classification, outside of enrichment for *BRAF* mutations and fewer *APC* mutations, in the PDS2 group (these are expected within the CMS1 and CMS4 groups, respectively), mutational and copy number profiles across all key genes assessed within the WNT, MAPK, PIK3CA, cell cycle, or TGF-β pathways were identical in PDS1 and PDS3, again, the two groups that contained equal proportions of CMS2 tumours. Downstream characterisation of PDS groups revealed that despite the absence of any genomic distinctions, these transcriptionally distinct subtypes were dominated by highly significant differences in many of the key cancer-associated hallmarks used in subtyping studies. As expected, PDS2 tumours were enriched for many traits associated with inflammatory/immune signalling pathways, such as stroma-related epithelial-to-mesenchymal transition (EMT), TGF-β pathway activation, and interferon responses. However, while PDS1 tumours displayed uniform and highly significant elevation of cell cycle-related pathways and MYC/WNT target activation in every single sample classified, there was almost universal transcriptional repression in PDS3 for many previously defined cancer-associated hallmarks [51].

Furthermore, while PDS1 was associated with fast-cycling canonical stem cells (LGR5 staining and CBC signatures), PDS2 was associated with regenerative stem cells (ANXA1 staining and RSC signatures), similar to the observed repression for cancer-relevant hallmarks; PDS3 was depleted/devoid of both of these stem populations and displayed low Ki67 staining. Although the majority of the previously described studies have focussed on changes in the stem populations, the absence of these cell populations was coupled with signalling in PDS3 tumours that appeared to indicate elevated numbers of differentiated colonic epithelial lineages, particularly transit-amplifying cells, enteroendocrine cells, and enterocytes (Fig. 4).

When H&Es were assessed either manually or using AI models (similar to imCMS), PDS3 tumours were indistinguishable from PDS1 and PDS2, and no pathological features or differentiation/grading differences were observed. While the presence of a slow-cycling, stem-depleted, and transcriptionally repressed group that is indistinguishable by histology and contains the same genomic profile as other tumours is interesting in itself, when tested in a series of clinical cohorts including the PETACC-3 clinical trial, PDS3 tumours represented the worst stage II/III prognostic group in terms of relapse-free survival following surgery, regardless of treatment.

To complement the PDS classifier, and the numerous stem cell classifiers that exist, we proposed a ‘Stem Maturation Index’ (SMI) classifier tool that provides a macro-view of overall cellular states when used in bulk data, or, when used in single-cell data, a comparative measurement of stem-ness versus differentiation for individual epithelial lineages. Importantly, this approach also offers a smoother transition between bulk, single-cell, and spatial transcriptomics, as it can reduce technical biases that undermine individual gene/probe assessments across platforms, enabling a more robust assessment of subtle signalling pathways underpinning tumour cell identity. While the previous stem cell classifiers may suggest that tumour cells display one or more of these stem states, our PDS and SMI data indicates that ~ 25–30% of CRC are more aligned to features of normal-like epithelial homeostasis in terms of stem-to-differentiated ratios even when they display all the same proportions of cancer driver mutations.

## Looking forward—re-discovery of findings from bulk and single-cell research in the era of spatial profiling

The use of bulk molecular data, which has been used here to describe any method that does not specifically sort different lineages prior to processing, typically involves the use of macro-dissected tissue from annotated slides, tissue curls or fresh/frozen tissue pieces. While estimates can be made about lineage abundance from annotated H&E or IHC-stained sequential sections if available, the precise composition of the tissue sample used to generate the bulk molecular data remains unknown. Furthermore, although estimates can be made as to these abundances, a reliable estimate of the identity of the precise lineage(s) that each RNA/DNA signal arises from cannot be determined, meaning that bulk profiles in each of these studies only offer, in the case of RNAseq, an average expression value for each gene across the full milieu of cells that were processed in each sample.

When discussed in this context, the advantages of single-cell technologies and the lineage-specific resolution they bring have offered the field an intriguing insight into the presence and extent of both genomic and transcriptomic heterogeneity within tumours. It can be argued that in the era of single-cell technologies, bulk profiling is too dated to be useful; however, as exemplified by the PDS study, bulk transcriptomics can still successfully be used as the basis for novel biological discovery and risk-stratification that can in-turn be interrogated/validated with newer methods. Furthermore, although the lack of lineage-specific information attributed to bulk profiling discussed above is a limitation, the fact that serial sections can be used to identify the precise localisation of expression in the same tumour has enabled bulk discoveries to provide some insights into subtyping/biomarker research and translational/diagnostic pathology.

The studies described here highlight how, as technologies advance, so too does our understanding of the intricate mechanisms underpinning cancer development and progression, revealing a unique insight into the sometime subtle signalling pathways that are likely to be key to the inter-compartmental crosstalk that drives tumour-wide responses. It could also be argued that many of the key findings from the subtyping studies over the last decade have relied heavily on molecular events and histological features that were previously discovered and characterised using routine histopathology and immunohistochemistry, as exemplified by the alignment between the Jass and CMS classifications, or the placement of well-established Vogelstein-described molecular events within previously defined polyp morphologies.

In line with this latter argument, in the same way that bulk profiling preceded single-cell technologies in biomarker development and molecular studies, the advent and widespread adoption of spatial-based technologies holds enormous potential for driving our understand on further. Future studies will likely begin to use parallel deep phenotyping methodologies in bulk and single-cell sequencing data from the same sample, complemented with advanced in situ tissue profiling using spatial transcriptomics, multiplex immunofluorescence/proteomics, alongside AI-based digital pathology. In this scenario, the signalling pathways and subtypes that were collapsed into an average score in bulk profiling could be revealed in individual cells at high-resolution across the entire field of cancer cells and stroma that pathologists use to generate diagnostic reports.

It may be unsurprising that a review such as this would end by promoting the value of pathology in guiding the next wave of molecular subtyping discoveries; however, the CRC field is on the cusp of producing some of the largest and most detailed tissue-based datasets that have ever existed. In this new era of spatially informed molecular research, pathology-led studies are once again required to ensure that cancer discoveries are developed based on the discipline that bridges science and medicine.

## Declarations

This review manuscript does not provide any original research findings, but only reviews previously published manuscripts and discusses their findings, placing them into perspective. Therefore, there is compliance with ethical standards through previous ethically acceptable publications.
