# Correction to: Molecular pathological classification of colorectal cancer—an update

**DOI:** 10.1007/s00428-024-03773-0

**Published:** 2024-02-28

**Authors:** Philip D. Dunne, Mark J. Arends

**Affiliations:** 1 Patrick G, Johnston Centre for Cancer Research, Queens University Belfast, Belfast, Northern Ireland BT8 7AE UK; 2 Cancer Research UK Scotland Institute, Garscube Estate, Glasgow, G61 1QH UK; 3 Edinburgh Pathology & Cancer Research UK Scotland Centre, Institute of Genetics & Cancer, University of Edinburgh, Crewe Road, Edinburgh, EH4 2XR UK

**Correction to: Virchows Archiv**

10.1007/s00428-024-03746-3

In the original published version of the above article, the reference Malla et al. (2024) was missing and should have been added within the 'Pathway-derived subtyping' section, alongside reference 51. The reference information should have appeared under the reference list as shown below.

**Reference**

52. Malla SB, Byrne RM, Lafarge MW et al (2024) Pathway level subtyping identifies a slow-cycling biological phenotype associated with poor clinical outcomes in colorectal cancer. Nat Genet. 10.1038/s41588-024-01654-5

